# Long term NIV in an infant with Hallermann-Streiff syndrome: A case report and overview of respiratory morbidity

**DOI:** 10.3389/fped.2022.1039964

**Published:** 2022-11-03

**Authors:** S Guerin, S Blanchon, Q de Halleux, V Bayon, T Ferry

**Affiliations:** ^1^ Unité de Pneumologie et Mucoviscidose Pédiatrique, Département Femme-Mère-Enfant, Centre Hospitalier Universitaire Vaudois et Université de Lausanne, Lausanne, Suisse; ^2^ Unité de Physiothérapie Pédiatrique, Département Femme-Mère-Enfant, Centre Hospitalier Universitaire Vaudois, Lausanne, Suisse; ^3^ Centre d’Investigation et de Recherche sur le Sommeil, Centre Hospitalier Universitaire Vaudois et Université de Lausanne, Lausanne, Suisse; ^4^ Soins Intensifs Pédiatriques, Département Femme-Mère-Enfant, Centre Hospitalier Universitaire Vaudois et Université de Lausanne, Lausanne, Suisse

**Keywords:** Hallermann-Streiff syndrome, NIV (non-invasive ventilation), OSAS (Obstructive sleep apnea syndrome), infant, success

## Abstract

Hallermann-Streiff syndrome (HSS) is a rare congenital syndrome with different anomalies including midface hypoplasia, beak nose and micrognathia. The upper airways narrowness can lead to severe respiratory complications such as obstructive sleep apnoea syndrome (OSAS), particularly in infancy. The management of these severe OSAS is difficult and poorly documented in literature. We report the case of an infant with HSS complicated by severe and early OSAS successfully managed with non-invasive ventilation (NIV), provide an overview of respiratory morbidities and discuss treatment options for HSS-related OSAS.

## Introduction

Hallermann-Streiff syndrome (HSS) is a rare congenital syndrome, first described by Francois in 1948 (1). To date, less than 200 individuals with a HSS have been described worldwide (2). A recent publication estimated its prevalence at 1/10 million in Japan (3). The phenotype is highly recognizable but genetic basis and molecular defect remain unknown (4). It is defined by the association of dyscephaly with midface hypoplasia, beak shape nose and micrognathia, proportional short stature, hypotrichosis, skin atrophy, dental abnormalities, microphthalmia, bilateral congenital cataracts and inconstant neurodevelopmental delay. Respiratory disorders are a major issue, especially during childhood. The upper airways narrowness leads to obstructive apneas, anesthetic management difficulties and potentially life-threatening events in severe cases (5). Obstructive Sleep Apnea Syndrome (OSAS) management in HSS is not well established and tracheostomy remains the chosen option when severe obstruction occurs (4). However, since last decades we face an important increase of non-invasive ventilation (NIV) use in children in several indications including OSAS related to facial anomalies (6).

We report the case of an infant with HSS complicated by severe and early OSAS, successfully managed with NIV. Furthermore, we give an overview of respiratory morbidities and discuss therapeutic options in OSAS related to HSS.

## Case

A female infant was born after 38 weeks of gestation of a normal pregnancy with birth parameters of 3110 g for the weight (percentile 25–50), length of 47 cm (percentile 5) and cranial circumference of 32.5 cm (percentile 5). She showed facial dysmorphic signs: tiny nose with marked nasal cartilage hypoplasia and skin atrophy, micro-retrognathy, microcephaly, neonatal teeth (51, 61, 71 and 81), bilateral congenital cataract, atrial septal defect and two small apical ventricular septal defects. HSS was strongly suspected, based on typical clinical phenotype. Genetic analyses (Next Generation Sequencing – NGS - of whole exome with analysis of 82 genes involved in early ageing and 1,832 genes involved in developmental disorders) were not able to find any causative genetic anomaly. First evaluation by Ear Nose and Throat (ENT) specialist through upper airways endoscopy at day 3 revealed narrow but permeable choanas without associated laryngo- trachea-malacia nor adenotonsillar hypertrophy. She was referred to pediatric pulmonologist because of high risk of apneas in this syndrome. First nocturnal oxymetry at day 4 and second at 5 months were normal. Successive peadiatric evaluations revealed a neurodevelopmental delay and a failure to thrive related to multifactorial feeding difficulties with inadequate intakes, daily aspirations and severe gastroesophageal reflux. At 8 months, she was admitted in hospital for investigations. She had an axial hypotonia (difficult head hold, no sitting) but a good grasp of objects and a good interaction despite the visual impairment. During the hospital stay, apneas were observed and the polygraphy confirmed a severe OSAS with an obstructive apnea-hypopnea index (OAHI) of 25/h and 0.4% of nighttime with SpO2 < 90%. Repeated venous blood gas showed no hypercapnia nor bicarbonate elevation. ENT endoscopy was repeated, showing still narrow choanas and adenoid hypertrophy, driving to adenoidectomy. At the same time, surgical gastrostomy, without anti-reflux surgery, was performed for enteral feeding considering the low weight (3,870 g, < 3 percentile).

**Figure 1:**
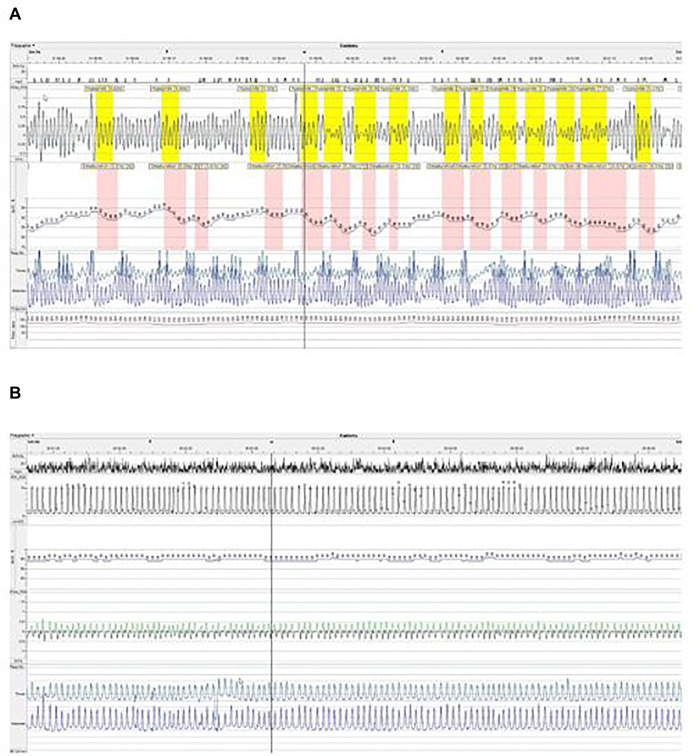
Polygraphy after adenotonsillectomy without NIV treatment (**A**) and under NIV treatment (**B**) in (**A**,**B**), we show extracts from polygraph plot with nasal flux (XFlow), oxygen saturation (SpO2), thoracic and abdominal movements and cardiac pulse. In (**A**), we see an obstructive breathing pattern with frequent hypopneas characterized by a drop of airflow associated with desaturations ≥3% of oxygen. In (**B**), under NIV, we see a regular breathing pattern with a complete disappearance of obstructive events and desaturation.

Six weeks later, snoring and disrupted sleep persisted when the polygraphy showed worsening of OSAS, with an OAHI of 140/h, and 41% of nighttime with SpO2 < 90% (Figure 1A). Because of the severe hypoplasia of face middle third, tracheostomy was suggested to treat this severe OSAS. After multidisciplinary discussions involving the parents, ENT and maxillofacial surgeons, intensivists, pulmonologists and physiotherapists, we first chose to try NIV therapy.

Nighttime NIV therapy was therefore initiated at 10-months-old (4,450 g/59,5 cm) in an intermediate care unit. Continuous positive airway pressure (CPAP) with a nasal mask was first started. Tolerance of CPAP was good but the polygraphy under CPAP showed no improvement of the OSAS. Increasing the level of Expiratory Positive Airway Pressure (EPAP) led to poor tolerance during expiratory time due to high level of pressure limiting expiratory flow and no additional gain on obstruction of the inspiratory flow. Therefore, we tried spontaneous bilevel positive pressure support (BPAP), using a Trilogy 100^©^ (Philips Respironics, Pennsylvania, USA) with single limb circuit and a nasal mask Respireo SOFT Baby^©^ (AirLiquide, France). EPAP and Inspiratory Positive Airway Pressure (IPAP) were subsequently modified to optimize inspiratory and expiratory flows as well as tolerance of NIV therapy and clinical respiratory pattern. After this titration phase, EPAP and IPAP were + 5 and + 9 cmH2O, respectively. Due to the risk of aspiration, enteral feeding was exclusively performed during daytime, with no overlap with NIV. Polygraphy under NIV, after 5 days of BPAP support, showed dramatic improvement of OSAS with an obstructive AHI of 7.6/h and only 1% of time with SpO2 < 90% (Figure 1B).

After training the parents regarding NIV and gastrostomy management, the patient was discharged at home. Respiratory situation remained good during the following year without any adverse event. The median time of respiratory support was 8 h overnight and no additional modification of NIV parameters was needed. Regular maxilla-facial evaluations showed no NIV-induced facial deformation. Parents reported rapid improvement in psychomotor acquisitions, interaction and general behavior.

One year later, polygraphy under NIV remained normal, with an OAHI of 1.4/h and less than 1% of time with SpO2 < 90%. To date, at 2 years old, the neuro-development was estimated equivalent to a 12-month-old child. Regular evaluations were made by a maxillo-facial surgeon. Facial evolution was as good as expected, with no clinical evidence of negative consequence of the nasal mask pressure on midface development.

## Discussion

Respiratory morbidity is important in HSS. It is highly variable but may potentially be life-threatening (7–10). Microretrognatia, microstome, and mid-face hypoplasia with narrow nose, sometimes associated with glossoptosis or laryngomalacia, result in an important narrowness of upper airways leading to anesthetic airway management difficulties (11) and OSAS (2). Tracheomalacia has also been reported in some cases of HSS (9), and tracheobronchoscopy should be performed in case of suggestive symptoms (early-onset inspiro-expiratory noise, fixed wheeze, recurrent infections, or chronic brassy cough). No published data or expert opinion supports that tracheomalacia management should be different in the context of HSS than in other conditions (12).

OSAS is definitely the main respiratory issue in HSS, but is challenging as no specific guidelines for OSAS screening and management in HSS is available. Therefore, physicians have to refer to general guidelines for OSAS in children (13, 14) or to experiences and expert recommendations in other craniofacial malformations (6, 15). Prevalence of OSAS in HSS is unknown, but may be high as reported in about half of the published cases. As in other craniofacial malformations, OSAS severity is highly variable between patients and is diagnosed at different ages (6), depending on the severity of facial anomalies and the possible association with other individual characteristics not related with HSS and influencing upper airways obstruction such as adenotonsillar hypertrophy or overweight. Regarding our patient, OSAS appeared during the first months of life (confirmed at 8 months) but in other HSS cases, OSAS was diagnosed later in childhood, even at adulthood (16). In syndromic cranio-synostosis such as Alpert or Crouzon syndrome, authors described an improvement of OSAS severity with growth (17), but no equivalent data are available in HSS.

In our case, severe OSAS was diagnosed at 8 months old by a polygraphy, when previous evaluations were normal, and therefore reassuring, but only based on nocturnal oxymetry. A rapid worsening of OSAS during the first months of life is possible, especially considering the role of adenoid growth in its physiopathology, but we can legitimately ask ourselves if the oxymetries did not underestimate apneas. Based on this experience, even if oximetry is more and more used for OSAS evaluation (18), we would recommend polygraphy [with measurement of nasal flow, thoracic and abdominal movements, heart rate and transcutaneous oxymetry (19)] for OSAS screening in babies with craniofacial malformations, to be able to detect OSAS as early as possible. The choice between polygraphy and polysomnography for OSAS evaluation can also be discussed. Even if the current gold standard for OSAS diagnostic in children remains polysomnography, it is now established in literature that polygraphy, because of its better accessibility and better feasibility especially for infants, is a good alternative to polysomnography for OSAS diagnosis (13).

Pediatric OSAS often requires a multidisciplinary management, especially in case of craniofacial malformations, including HSS. In children with craniofacial malformations, such as in all children, adenotonsillectomy must be provided in case of obstructive adenotonsillar hypertrophy, as we did for our patient (13). Unfortunately in this case, OSAS persisted after this first surgical step. There is no available data on adenotonsillectomy efficacy for OSAS treatment in HSS but it is widely reported in other craniofacial malformations that OSAS can persist after adenotonsillectomy, especially if initial OSAS was severe (6, 13). Therefore, following ERS recommendations for OSAS management in children (13), an assessment by polysomnography or polygraphy should be systematically performed after adenotonsillectomy in HSS population.

If adenotonsillectomy is not indicated or in case of persistent severe OSAS after adenotonsillectomy, the second line of treatment must be carefully discussed in a multidisciplinary team. In our case, regarding the severity of OSAS, the potential negative impact on growth and neurodevelopmental status and the high risk of life-threatening obstructive event, an additional treatment with short-term efficacy was mandatory. Maxillofacial surgery, tracheostomy and non-invasive positive pressure support are the three main therapeutic options to discuss in order to find the personalized most appropriate strategy.

– *Maxillo-facial surgery*: Several surgical options have been proposed for HSS, depending on the severity and location of the middle third hypoplasia (20, 21). These surgical treatments appear to be efficient for OSAS treatment, knowing that reconstructive surgery are usually proposed at an adult's age or in late adolescence, as midface advancement required a mature facial ossification. In case of severe obstruction early in life, as experienced by our patient, it was not considered as a short-term option.
– *Tracheotomy:* Several cases of HSS undergoing tracheostomy have been reported, with resolution of the OSAS (8). This is the most radical option to bypass upper airway obstruction, and the chosen treatment in main of the published cases with HSS-related OSAS. This option should be considered after careful evaluation considering the consequences on orality, speech development and sociability, and the potential risk of complications (infectious complications, long-term tracheal stenosis or granulation, life-threatening decannulation or cannula obstruction events).
– *Non-Invasive Ventilation (NIV) therapy:* NIV (including– CPAP and–BPAP) is a validated treatment of OSAS in children if ENT and/or maxillofacial surgical options are impossible or ineffective (13). To our knowledge, there is no published case of HSS children with severe OSAS treated by NIV to date, but in other craniofacial malformations, it is now a well-established therapeutic option (6), with good efficiency on OSAS and good tolerance. However, some difficulties should be mentioned. First, finding an appropriate interface can be challenging because of the paucity of available masks suitable for young children. This is even more challenging in HSS babies given their specific facial features. Second, the risk of broncho-inhalation in case of reflux or vomiting should also be evaluated, especially in young children. Third, the impact of mask pressure on the face development has to be discussed and monitored if used for a long time. Finally, depending of the severity of the obstruction, high pressure level could be required which can be poorly tolerated by the child.

In our case, the risk of NIV intolerance was relatively high, considering the severe midface hypoplasia which reduce the choice of appropriate interface, the narrowing of airways at different stages (i.e., nares-choanas-oropharyngeal), the severe gastro-oesophageal reflux with risk of aspiration, and the small weight with possible ventilation asynchronism. Despite this, we decided to try NIV therapy for our patient, which was considered by the team and parents as the less invasive and the more appropriate option.

So far both the parents and the multidisciplinary medical team remain convinced that NIV was the good therapeutical choice for this child. NIV corrected OSAS, helped the patient to grow and to continue her neurodevelopmental progress, being the bridge to maxillofacial - hopefully curative—surgery in several years and avoiding the need of tracheostomy.

In our opinion, the success of NIV in this case was multifactorial. The multidisciplinary approach, including paediatric pulmonology, intensive care and ENT specialists, maxillo-facial surgeon, physiotherapist, and a well-experienced team in NIV in children with access to appropriate devices, allowed a careful and global evaluation of the feasibility and a fine tuning of the NIV device. Nevertheless, the success of NIV in such cases would not be possible without very motivated and appropriate parents. Obviously, close follow-up is mandatory during NIV therapy in order to detect any NIV adverse event, to evaluate adequacy of NIV support and OSAS evolution, performing iterative polygraphy, which we believe should be performed at least once a year during NIV treatment. In this context of facial congenital malformation, regular evaluations by maxillo-facial surgeon are mandatory to properly assess NIV impact on mid-face growth.

This success well illustrates that NIV should be considered in patients with HSS and OSAS, such as in other craniofacial malformations like Treacher Collins, Pierre-Robin or Alpert Syndrome.

## Conclusion

OSAS is the main respiratory issue in HSS, mainly due to facial malformations. Screening for OSAS should be systematic within the first months of life and repeated during childhood, preferably by polygraphy or polysomnography. NIV could be considered as a safe and feasible option for OSAS treatment, as an alternative to early tracheostomy, in addition or as a bridge to ENT and/or maxillofacial surgery.

## Data Availability

The original contributions presented in the study are included in the article/Supplementary Material, further inquiries can be directed to the corresponding author/s.
