# Erratum: Long term NIV in an infant with Hallermann-Streiff syndrome: A case report and overview of respiratory morbidity

**DOI:** 10.3389/fped.2023.1173254

**Published:** 2023-03-07

**Authors:** Frontiers Production Office

**Affiliations:** Frontiers Media SA, Lausanne, Switzerland

**Keywords:** Hallermann-Streiff syndrome, NIV (non-invasive ventilation), OSAS (Obstructive Sleep Apnea Syndrome), infant, success

An Erratum on Long term NIV in an infant with Hallermann-Streiff syndrome: A case report and overview of respiratory morbidity By Guerin S, Blanchon S, de Halleux Q, Bayon V and Ferry T. (2022) Front. Pediatr. 10:1039964. doi: 10.3389/fped.2022.1039964

An omission to the funding section of the original article was made in error. The following sentence has been added: “Open access funding was provided by University of Lausanne”.

The original version of this article has been updated.

