# Corrigendum: Long term NIV in an infant with Hallermann-Streiff syndrome: a case report and overview of respiratory morbidity

**DOI:** 10.3389/fped.2024.1521164

**Published:** 2024-12-05

**Authors:** S. Guerin, S. Blanchon, Q. de Halleux, V. Bayon, T. Ferry

**Affiliations:** ^1^ Unité de Pneumologie et Mucoviscidose Pédiatrique, Département Femme-Mère-Enfant, Centre Hospitalier Universitaire Vaudois et Université de Lausanne, Lausanne, Suisse; ^2^ Unité de Physiothérapie Pédiatrique, Département Femme-Mère-Enfant, Centre Hospitalier Universitaire Vaudois, Lausanne, Suisse; ^3^ Centre d’Investigation et de Recherche sur le Sommeil, Centre Hospitalier Universitaire Vaudois et Université de Lausanne, Lausanne, Suisse; ^4^ Soins Intensifs Pédiatriques, Département Femme-Mère-Enfant, Centre Hospitalier Universitaire Vaudois et Université de Lausanne, Lausanne, Suisse

**Keywords:** Hallermann-Streiff syndrome, NIV (non-invasive ventilation), OSAS (Obstructive sleep apnea syndrome), infant, success

A Corrigendum on Long term NIV in an infant with Hallermann-Streiff Syndrome: a case report and overview of respiratory morbidity By Guerin S, Blanchon S, de Halleux Q, Bayon V, Ferry T. (2022) Front Pediatr. 10:1039964. doi: 10.3389/fped.2022.1039964

Error in Figure:

In regard to Figure 2, the parents of the child depicted in the photos provided their written consent for publication in 2022. However, they now wish to have the photos removed from the article because they have been indexed by general search engines and are appearing on the internet beyond specialized medical websites.

On behalf of the parents, and with the agreement of all authors, we respectfully request the removal of Figure 2 (including the photos and caption) from the article.

Text Correction:

Dut to the removal of Figure 2, citation relatives to Figure 2 have been removed from the text.

A correction has been made to the **Case** section.

This sentence previously stated:

“Figure 2 displays photos of the patient with and without NIV.”

This sentence has now been removed.

